# Dairy consumption and risks of total and site-specific cancers in Chinese adults: an 11-year prospective study of 0.5 million people

**DOI:** 10.1186/s12916-022-02330-3

**Published:** 2022-05-06

**Authors:** Maria G. Kakkoura, Huaidong Du, Yu Guo, Canqing Yu, Ling Yang, Pei Pei, Yiping Chen, Sam Sansome, Wing Ching Chan, Xiaoming Yang, Lei Fan, Jun Lv, Junshi Chen, Liming Li, Timothy J. Key, Zhengming Chen, on behalf of the China Kadoorie Biobank (CKB) Collaborative Group, Junshi Chen, Zhengming Chen, Robert Clarke, Rory Collins, Yu Guo, Liming Li, Chen Wang, Jun Lv, Richard Peto, Robin Walters, Daniel Avery, Derrick Bennett, Ruth Boxall, Ka Hung Chan, Yumei Chang, Yiping Chen, Johnathan Clarke, Huaidong Du, Zammy Fairhurst-Hunter, Hannah Fry, Simon Gilbert, Alex Hacker, Mike Hill, Michael Holmes, Pek Kei Im, Andri Iona, Maria Kakkoura, Christiana Kartsonaki, Rene Kerosi, Kuang Lin, Mohsen Mazidi, Iona Millwood, Qunhua Nie, Alfred Pozarickij, Paul Ryder, Saredo Said, Sam Sansome, Dan Schmidt, Paul Sherliker, Rajani Sohoni, Becky Stevens, Iain Turnbull, Lin Wang, Neil Wright, Ling Yang, Xiaoming Yang, Pang Yao, Xiao Han, Can Hou, Qingmei Xia, Chao Liu, Pei Pei, Canqing Yu, Naying Chen, Duo Liu, Zhenzhu Tang, Ningyu Chen, Qilian Jiang, Jian Lan, Mingqiang Li, Yun Liu, Fanwen Meng, Jinhuai Meng, Rong Pan, Yulu Qin, Ping Wang, Sisi Wang, Liuping Wei, Liyuan Zhou, Caixia Dong, Pengfei Ge, Xiaolan Ren, Zhongxiao Li, Enke Mao, Tao Wang, Hui Zhang, Xi Zhang, Jinyan Chen, Ximin Hu, Xiaohuan Wang, Zhendong Guo, Huimei Li, Yilei Li, Min Weng, Shukuan Wu, Shichun Yan, Mingyuan Zou, Xue Zhou, Ziyan Guo, Quan Kang, Yanjie Li, Bo Yu, Qinai Xu, Liang Chang, Lei Fan, Shixian Feng, Ding Zhang, Gang Zhou, Yulian Gao, Tianyou He, Pan He, Chen Hu, Huarong Sun, Xukui Zhang, Biyun Chen, Zhongxi Fu, Yuelong Huang, Huilin Liu, Qiaohua Xu, Li Yin, Huajun Long, Xin Xu, Hao Zhang, Libo Zhang, Jian Su, Ran Tao, Ming Wu, Jie Yang, Jinyi Zhou, Yonglin Zhou, Yihe Hu, Yujie Hua, Jianrong Jin Fang Liu, Jingchao Liu, Yan Lu, Liangcai Ma, Aiyu Tang, Jun Zhang, Liang Cheng, Ranran Du, Ruqin Gao, Feifei Li, Shanpeng Li, Yongmei Liu, Feng Ning, Zengchang Pang, Xiaohui Sun, Xiaocao Tian, Shaojie Wang, Yaoming Zhai, Hua Zhang, Wei Hou, Silu Lv, Junzheng Wang, Xiaofang Chen, Xianping Wu, Ningmei Zhang, Weiwei Zhou, Jianguo Li, Jiaqiu Liu, Guojin Luo, Qiang Sun, Xunfu Zhong, Weiwei Gong, Ruying Hu, Hao Wang, Meng Wan, Min Yu, Lingli Chen, Qijun Gu, Dongxia Pan, Chunmei Wang, Kaixu Xie, Xiaoyi Zhang

**Affiliations:** 1 Clinical Trial Service Unit and Epidemiological Studies Unit (CTSU), Nuffield Department of Population Health, University of Oxford, Oxford, UK; 2 Medical Research Council Population Health Research Unit (MRC PHRU), Nuffield Department of Population Health, University of Oxford, Oxford, UK; 3 Fuwai Hospital Chinese Academy of Medical Sciences, National Center for Cardiovascular Diseases, Beijing, China; 4 Department of Epidemiology and Biostatistics, School of Public Health, Peking University, Beijing, China; 5 Chinese Academy of Medical Sciences, Beijing, China; 6 NCDs Prevention and Control Department, Henan CDC, Zhengzhou, China; 7 China National Center for Food Safety Risk Assessment, Beijing, China; 8 Cancer Epidemiology Unit (CEU), Nuffield Department of Population Health, University of Oxford, Oxford, UK

**Keywords:** Cancer, Dairy products, Diet, China, Prospective cohort study

## Abstract

**Background:**

Previous studies of primarily Western populations have reported contrasting associations of dairy consumption with certain cancers, including a positive association with prostate cancer and inverse associations with colorectal and premenopausal breast cancers. However, there are limited data from China where cancer rates and levels of dairy consumption differ importantly from those in Western populations.

**Methods:**

The prospective China Kadoorie Biobank study recruited ~0.5 million adults from ten diverse (five urban, five rural) areas across China during 2004–2008. Consumption frequency of major food groups, including dairy products, was collected at baseline and subsequent resurveys, using a validated interviewer-administered laptop-based food frequency questionnaire. To quantify the linear association of dairy intake and cancer risk and to account for regression dilution bias, the mean usual consumption amount for each baseline group was estimated via combining the consumption level at both baseline and the second resurvey. During a mean follow-up of 10.8 (*SD* 2.0) years, 29,277 incident cancer cases were recorded among the 510,146 participants who were free of cancer at baseline. Cox regression analyses for incident cancers associated with usual dairy intake were stratified by age-at-risk, sex and region and adjusted for cancer family history, education, income, alcohol intake, smoking, physical activity, soy and fresh fruit intake, and body mass index.

**Results:**

Overall, 20.4% of participants reported consuming dairy products (mainly milk) regularly (i.e. ≥1 day/week), with the estimated mean consumption of 80.8 g/day among regular consumers and of 37.9 g/day among all participants. There were significant positive associations of dairy consumption with risks of total and certain site-specific cancers, with adjusted HRs per 50 g/day usual consumption being 1.07 (95% *CI* 1.04–1.10), 1.12 (1.02–1.22), 1.19 (1.01–1.41) and 1.17 (1.07–1.29) for total cancer, liver cancer (*n* = 3191), female breast cancer (*n* = 2582) and lymphoma (*n*=915), respectively. However, the association with lymphoma was not statistically significant after correcting for multiple testing. No significant associations were observed for colorectal cancer (*n* = 3350, 1.08 [1.00–1.17]) or other site-specific cancers.

**Conclusion:**

Among Chinese adults who had relatively lower dairy consumption than Western populations, higher dairy intake was associated with higher risks of liver cancer, female breast cancer and, possibly, lymphoma.

**Supplementary Information:**

The online version contains supplementary material available at 10.1186/s12916-022-02330-3.

## Background

Cancer is one of the leading causes of adult mortality globally [1]. In China, there are >4 million new cancer cases and ~3 million cancer deaths annually, with lung, colorectal, stomach, liver and female breast cancers being the most frequently diagnosed cancers [2]. Major risk factors for cancer include smoking, chronic infection, female reproductive factors, alcohol consumption and, potentially, certain dietary factors, including low intakes of fresh fruit, vegetables and whole grains and high intake of processed meat [3–5].

Several large prospective studies, including meta-analyses of these studies, of primarily Western populations have examined the associations of dairy intake with cancer risks, showing an inverse association with colorectal cancer [6], positive association with risk of prostate cancer [7] but lack of clear associations with risks of breast [8] and many other site-specific cancers [9]. Based on the available evidence, the World Cancer Research Fund/American Institute for Cancer Research (WCRF/AICR) concluded that although the overall evidence on the dairy and cancer relationship is still inconsistent, there is strong evidence supporting the probable protective role of dairy products in the incidence of colorectal cancer and limited evidence suggesting that higher dairy intake is associated with higher prostate cancer risk [5].

Few prospective studies have been conducted in non-Western populations [5], among which cancer rates [1], the type and average amount of dairy consumption [10, 11] and genetic ability to metabolize dairy products differed greatly from those in Western countries [12]. In China, although the per capita consumption of milk has increased during recent decades, it is still far lower than in Europe (32.7 vs. 215.1 kg/year in 2013) [13]. In addition, cheese is hardly consumed [14, 15] and a large majority of the population cannot properly digest lactose [12]. To date, the few prospective studies from China on the topic showed no significant associations linking dairy intake with risks of female breast or colorectal cancers [16, 17].

To fill in the knowledge gap, we investigated the associations of habitual dairy consumption with total and site-specific cancer incidence in the China Kadoorie Biobank (CKB) study, a large nationwide prospective cohort study of Chinese adults. As a secondary objective, we also assessed whether potential cancer-related risk factors, such as sociodemographic and lifestyle factors and adiposity [1, 4], could modify the associations between dairy intake and cancer incidence.

## Methods

### Study population

The CKB is a population-based prospective study with over 0.5 million adults recruited from ten geographically diverse regions in China, with the majority of the participants being Han Chinese (~97%) and all of them speaking the Chinese language. The five urban regions included Qingdao (in Shandong Province), Harbin (in Heilongjiang Province), Haikou (in Hainan Province), Suzhou (in Jiangsu Province) and Liuzhou (in Guangxi Zhuang Autonomous Region) and the five rural regions were in Sichuan, Gansu, Henan, Zhejiang and Hunan Provinces. The study design and methods have been previously described elsewhere [18]. Briefly, these ten regions were chosen to cover a wide range of risk exposures and disease patterns. Between June 2004 and July 2008, all residents aged 35–74 years in preselected rural villages or urban residential committees were invited to participate in the study. About one in three (33% in rural areas and 27% in urban areas) responded and were enrolled in the study. In total, 512,726 participants were included, including 3.3% of them (i.e. with 9817 <35 years old and 7283 >74 years old) who were just outside the pre-specified target age range (therefore the actual baseline age ranged from 30 to 79 years), and they all provided informed consent. The study protocol and standard operating procedures have been applied to all participants. Ethics approvals were obtained at local, national and international levels prior to the beginning of recruitment.

### Baseline data collection

At each local study assessment clinic, trained health professionals administered a laptop-based questionnaire collecting information on sociodemographic characteristics, medical history and lifestyle factors. Anthropometrics were measured using standard protocols and a venous blood sample was collected from each participant for long-term storage. On-site measurement of hepatitis B surface antigen (HBsAg) was performed (ACON Biotech).

### Assessment of dietary intake

Information on consumption frequency (five categories including daily, 4–6 days/week, 1–3 days/week, monthly or never/rarely) of 12 major food groups (i.e. rice, wheat products, coarse grain products, red meat, poultry, fish, eggs, total dairy products, fresh vegetables, preserved vegetables, fresh fruit and soybean products) [3] was obtained using a validated interviewer-administered laptop-based questionnaire. This questionnaire aimed to record the habitual consumption frequency of major food groups by the participants over the preceding year. A separate validation study was performed among 432 CKB participants during 2015–2016 to assess the reproducibility, and validity of the questionnaire, using 12-day 24-hour recalls as reference (19). The results supported a good reproducibility and relative validity of total dairy consumption frequency, with the weighted kappa statistics being 0.82 and 0.75 for reproducibility and validity, respectively. In addition, the reproducibility and validity of all other dietary variables (mentioned above) were good as well, with the weighted kappa values being >0.60 for all, and the Spearman correlation coefficients for the validity of milk, yoghurt and other dairy products being 0.43, 0.36 and 0.31, respectively [19].

After completion of the baseline survey, two resurveys were undertaken in 5–6% of randomly selected surviving participants, with procedures largely identical to those at baseline. The data from these resurveys allow us to control for regression dilution bias when examining the prospective associations between baseline exposures and subsequent disease incidence or mortality in the whole population, by estimating long-term usual levels of exposures (i.e. dairy consumption) [20]. The second resurvey, performed from August 2013 to September 2014 among ~25,000 participants, also collected more detailed dietary data, including daily portions of each food group and the consumption (both frequency and amount) of three subtypes of dairy products, i.e. cow milk, yoghurt and other dairy products (e.g. cheese and milk powder). That information was used to estimate the mean usual amount of consumption (i.e. average level of dietary intake during the follow-up period) for each category of food consumption variable at baseline, including total dairy consumption (Additional file 1: Methods and Additional file 1: Table 1).

### Follow-up for incident cancer

The vital status of participants (including causes of deaths) was determined based on information from the Disease Surveillance Points system in China [21], checked annually against local residential records and health insurance records and confirmed by street committees or village administrators. Furthermore, information on cancer incidence was obtained through linkages via unique personal identification with cancer registries and nationwide health insurance claim databases, which provide electronic linkage to almost all hospitalizations (~99% coverage across the ten study regions) for CKB participants. These were supplemented by annual active follow-up of uninsured participants (~1%) to minimize any underreporting and ensure that the false-negative rate in cancer reporting is properly controlled [22]. In addition, the ongoing cancer outcome adjudication in a subset of cancer cases (*n* = 16,998 by 1 January 2018) via review of medical notes showed a ~90% reporting accuracy of primary diagnosis. Fatal and non-fatal events were International Classification of Diseases (ICD)-10 coded by trained staff, who were blinded to baseline information [18]. In the present study, we analysed the incidence of all cancers (ICD-10 C00-C97) and 17 common cancer sites (Additional file 1: Table 2). Only the first incident cancer diagnosis of each type was counted.

### Statistical analysis

Participants with a baseline history of cancer (*n* = 2578) or those with missing values for body mass index (BMI) (*n* = 2) were excluded, leaving 510,146 participants for analyses.

Multiple linear regression (for continuous outcomes) or logistic regression (for binary outcomes) were used to calculate the means (standard deviations (SDs)) or percentages of various baseline characteristics across three frequency categories of dairy consumption (i.e. never/rarely, monthly and ≥1 day/week-characterized as regular), with adjustments for sex, age and region where appropriate. Cross-sectional associations of dairy consumption with BMI, body weight, standing height, leg length, waist circumference (WC) and body fat percentage (BF%) in each sex group were analysed using multiple linear regression, adjusting for age, region, education, annual household income, smoking, alcohol consumption, total physical activity and fresh fruit consumption. Analyses for WC and BF% were additionally adjusted for BMI. A similar analysis was run, among those who participated in the second resurvey (*n* = 18,132), to examine the associations of dairy consumption (three frequency consumption categories) with changes in standing height, leg length, body weight and BMI.

Cox regression was used to calculate hazard ratios (HRs) and 95% confidence intervals (CIs) for cancer incidence in relation to baseline frequency categories of dairy consumption, stratified by age-at-risk (in 5-year intervals), sex, and region and adjusted for the aforementioned potential confounders (i.e. education, annual household income, smoking, alcohol consumption, total physical activity and fresh fruit consumption) plus family history of cancer, soy consumption and, for liver cancer only, status of HBsAg. We adjusted only for two dietary variables, fresh fruit and soybean, because fresh fruit intake was previously shown to be associated with cancer risk [3] and associations of dairy milk consumption with cancer risk may be confounded by soymilk consumption [23]. No other dietary variables played a confounding role in the current analysis including fresh vegetables (cooked and raw-eaten fresh vegetables but no preserved vegetables) for which there was an extremely high consumption frequency across all study areas (with 95% of participants reporting daily consumption). Participants were classified into three aforementioned frequency categories of dairy consumption to ensure adequate numbers of cancer cases in each consumption category. The proportional hazards assumption was assessed by comparing the HRs for the first and second half of the follow-up period, but no violation was observed. Participants who had died (~9%) or were lost to follow-up (~1%) were censored in the prospective analyses (censoring date was 1 January 2018). The floating absolute risk method, which provides variance of log risk for each category (including the reference group), was used to facilitate comparisons between any two exposure groups rather than just with an arbitrarily chosen reference group [24]. The HRs (95% CIs) for each 50-g/day increment in usual dairy consumption were calculated using Cox regression analyses (with same covariates mentioned above) to quantify the linear association and to correct for regression dilution bias [20] (see Additional file 1: Methods and Additional file 1: Table 1 on calculation of usual amount of dairy consumption)*.* Stratified analyses by potential effect modifiers, such as age-at-risk, region, smoking and adiposity, were carried out and chi-square tests were used to examine the significance of a trend or heterogeneity test. The Benjamini-Hochberg method was used to account for multiple comparisons and both unadjusted *P* values and false discovery rate (FDR)-corrected *P* values were reported (at 5% FDR).

Sensitivity analyses were performed by excluding the first 2 years of follow-up, excluding participants with baseline age below 35 and above 74 years and additionally adjusting for other covariates, including baseline prevalent cardiovascular disease and diabetes, other dietary variables, anthropometric factors and other potential risk factors for specific cancer types (e.g. baseline history of chronic hepatitis/cirrhosis for liver cancer and female reproductive factors for breast cancer). In addition, the analyses for breast cancer were also re-run after excluding women who reported a prior history of lumpectomy at baseline. The small numbers of participants with missing values for any of the variables used in the analytical models were omitted from the analyses. The statistical packages SAS (version 9.4, SAS Institute, Cary, NC, USA) and R 3.6.3 (https://www.R-project.org/) were used for performing the analyses and creating the figures.

## Results

Among the study participants, the mean (SD) baseline age was 52 (10.7) years, 59% were women and 44% resided in urban areas (Table 1). Overall, 20.4% reported consuming dairy at least once per week (defined as regular consumers hereafter), and 68.5% reported never or rare consumption (defined as non-consumers hereafter). Regular consumers were more likely to be women, had higher education and income levels and were less likely to report poor self-rated health. Moreover, regular dairy consumers were more likely to have a higher consumption of all major foods, except preserved vegetables. There was also a slightly higher proportion of regular consumers who self-reported prior history of cardiovascular disease or diabetes than non-consumers. The percentages of participants who had a positive HBsAg status and the mean values of total physical activity (metabolic equivalent-h/day) were similar across the three dairy consumption groups. In men, regular consumers were less likely to be smokers or alcohol drinkers (Table 1). In women, regular consumers reported a slightly higher proportion of oral contraceptive use and a slightly shorter mean breastfeeding duration (Additional file 1: Table 3).

**Table 1:** Characteristics of participants by frequency of dairy intake at baseline survey (2004–2008)

Characteristic	Frequency of dairy intake			Overall *(n* = 510,146)
	Never/rarely	Monthly	Regular	
	(*n* = 349,325)	(*n* = 56,750)	(*n* = 104,071)	
Usual dairy intake, g/day^a^	24.0	44.4	80.8	37.9
Mean age (SD), years	51.9 (11.1)	51.7 (10.8)	52.5 (11.8)	52.0 (10.7)
Women, %	58.3	57.5	62.1	59.0
Urban, %	30.8	54.2	83.0	44.1
Education >6 years, %	42.2	54.9	69.7	49.2
Household income >20,000 yuan/year, %	36.0	50.9	60.8	42.7
Ever regular smoking in men, %	76.0	73.7	68.6	74.3
Ever regular smoking in women, %	3.6	3.1	2.2	3.2
Ever regular alcohol drinking in men, %	38.6	34.6	32.8	37.0
Ever regular alcohol drinking in women, %	2.3	2.3	3.1	2.5
Frequency of food intake, %^b^				
Red meat	44.9	48.6	54.3	47.2
Poultry	23.6	30.1	42.7	28.2
Fish	7.8	9.2	12.5	8.9
Eggs	20.3	26.5	37.1	24.4
Fresh fruit	21.9	28.5	48.9	28.2
Fresh vegetables	94.4	93.9	96.4	94.8
Preserved vegetables	24.5	19.4	18.0	22.6
Soy products	8.0	13.2	14.4	9.9
Coarse grain products	12.9	12.3	18.4	13.9
Rice	71.1	71.8	73.4	71.7
Wheat products	44.0	47.6	57.7	47.2
Mean total physical activity (SD), MET-h/day	21.4 (12.4)	20.8 (12.1)	20.4 (13.3)	21.1 (13.9)
Mean BMI (SD), kg/m^2^	23.7 (3.4)	23.6 (3.3)	23.4 (3.6)	23.7 (3.4)
HBsAg positive, %^c^	3.1	3.1	3.1	3.1
Prevalent cardiovascular disease, %^d^	4.1	4.9	5.5	4.5
Prevalent diabetes, %^e^	5.4	6.3	7.4	5.9
Self-rated poor health, %	10.4	10.4	9.7	10.2

Multiple linear regression (for continuous outcomes) or logistic regression (for binary outcomes) was used to calculate the means (SDs) or percentages of various baseline characteristics across three frequency categories of dairy consumption (i.e. never/rarely, monthly and ≥1 day/week-characterized as regular), with adjustments for age (continuous), sex (dichotomous variable) and region (ten regions), where appropriate

*BMI* body mass index, *HBsAg* hepatitis B surface antigen, *MET* metabolic equivalent of task hours, *SD* standard deviation

^a^Crude mean values from the second resurvey (2013–2014) of randomly selected 24,700 participants without cancer at either baseline or second resurvey

^b^Percentage values indicate the frequency intake by the participants as ‘daily’ for fresh vegetable intake; ‘≥1 day/week’ for poultry intake and ‘≥4 days/week’ (i.e. ‘regular’) for all other food groups

^c^Values for HBsAg status were missing for 8159 participants

^d^Including participants with self-reported prior history of either chronic heart disease, stroke or transient ischemic attack

^e^Including participants with either screen-detected or self-reported prior history of physician-diagnosed diabetes

**Fig. 1:**
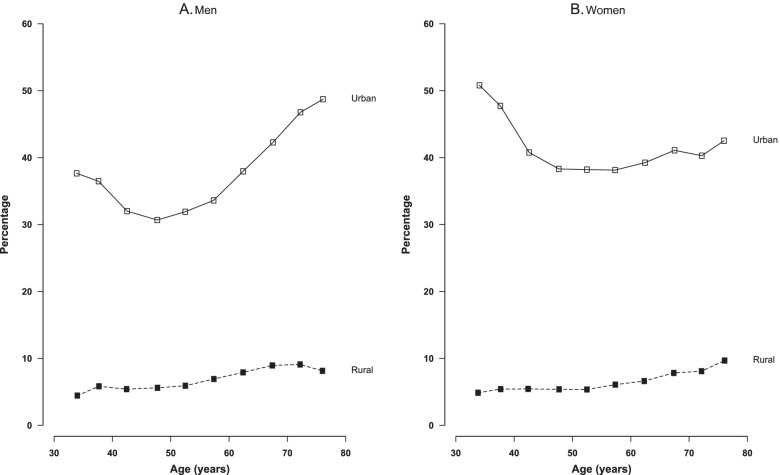
Percentage of men and women who reported regular dairy intake by age and study area at baseline (2004–2008)

In both sexes, dairy consumption was higher in urban than in rural areas. Across ten regions, the proportion of regular dairy consumers varied nearly 30-fold in both sexes (Additional file 1: Fig. 1). The estimated usual mean dairy consumption (37.9 g/day overall and 80.8 g/day in regular consumers) followed a similar ranking order (Additional file 1: Fig. 2). Baseline dairy consumption had a U- or L-shaped age pattern in both urban men and women, with the lowest proportion of regular consumers at 50 to 55 years old. However, in rural areas, the consumption increased gradually with age (Fig. 1).

**Fig. 2:**
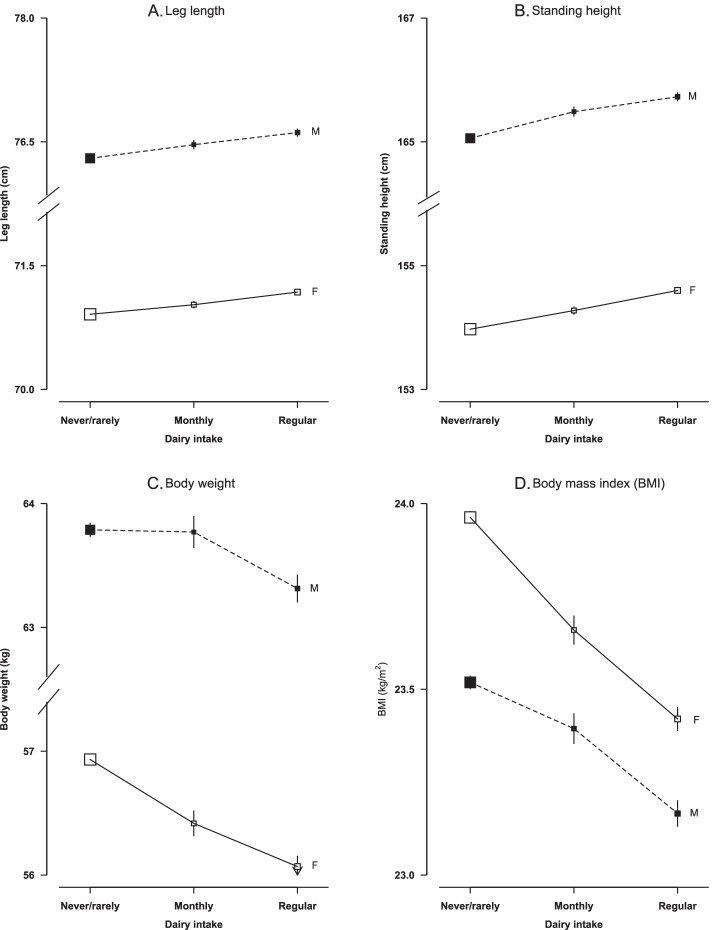
Adjusted mean leg length, standing height, body weight and body mass index by frequency of dairy intake in men (M) and women (F) at baseline (2004–2008). Linear regression analyses were adjusted for age (continuous variable), region (ten regions), education (four categories), annual household income (four categories), smoking (four categories), alcohol consumption (four categories), total physical activity (continuous variable) and fresh fruit consumption (five categories). Vertical lines represent 95% CIs. Solid squares represent men and open squares represent women

Standing height and leg length were both positively associated with dairy consumption in both sexes, with regular consumers having 0.6 cm higher standing height and 0.3 cm higher leg length than non-consumers. Body weight and BMI were both inversely associated with dairy consumption, with regular consumers being 0.5/0.9 kg and 0.4/0.5 kg/m^2^ lighter than non-consumers in males and females, respectively (Fig. 2). In addition, higher dairy consumption was related with a smaller height loss, less weight gain and lower BMI increase than in non-consumers, although the differences were rather small (Additional file 1: Fig. 3). No clear association was observed of dairy consumption with either WC or BF% (data not shown).

**Fig. 3:**
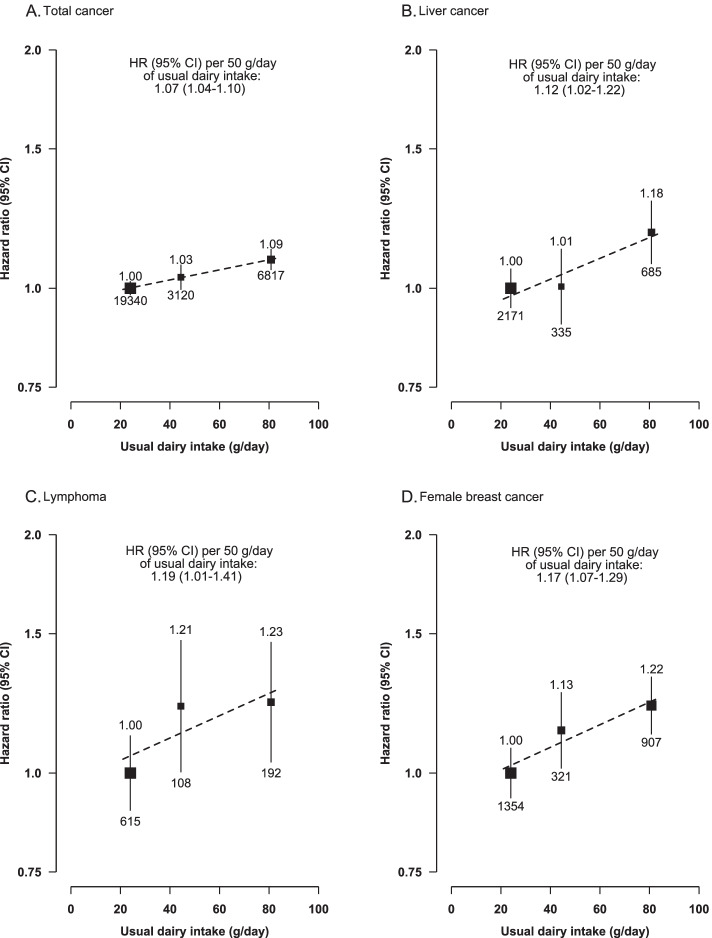
Associations of usual dairy intake (g/day) with the incidence of total cancer, liver cancer, lymphoma and female breast cancer. Cox regression analyses were performed among 510,146 participants with no prior self-reported history of cancer at baseline. Analyses were stratified by age-at-risk (continuous), sex (dichotomous variable) and region (ten regions) and were adjusted for education (four categories), income (four categories), smoking (four categories), alcohol consumption (four categories), total physical activity (continuous variable), family history of cancer (dichotomous), fresh fruit consumption (five categories), soy consumption (three categories) and BMI (continuous variable). **B** Analysis for liver cancer was additionally adjusted for HBsAg status (three categories). The *y* axis was plotted on a log_e_ scale with the lowest intake group (never/rarely) as a reference category. The estimated crude mean values of usual dairy intake (g/day) were 24.0, 44.4 and 80.8 g/day in the lowest (never/rarely), medium (monthly) and highest (regular) intake groups, respectively. The FDR-corrected *P* trend values for the associations with incidence of total cancer, liver cancer, lymphoma and female breast cancer were 0.002, 0.04, 0.17 and 0.01, respectively. The black squares represent HRs with the size being inversely proportional to the variance of the log_e_ of HR and the vertical lines represent 95% CIs. The numbers above the vertical lines are point estimates for HRs and the numbers below the lines refer to the number of incident cancer cases. Dashed diagonal lines indicate the linear associations between dairy intake and cancer risk

During a mean follow-up of 10.8 (SD 2.0) years and approximately 5.4 million person-years, a total of 29,277 incident cancer cases were recorded at ages 35–79 years (incidence rate 5.47 per 1000 person-years), with lung cancer having the highest incidence rate in the total population, followed by cancers of the female breast, stomach, colorectum and liver (Additional file 1: Table 2). After adjusting for the various aforementioned covariates, dairy consumption was significantly and positively associated with the risk of total cancer, with the adjusted HR of 1.09 (95% *CI* 1.06–1.12) when comparing regular with non-consumers. In addition, regular dairy consumption was also significantly associated with 18% (1.18; 1.08–1.29) and 22% (1.22; 1.12–1.32) higher risks of liver cancer and female breast cancer, respectively, in comparison with the non-consumers (Fig. 3). All these associations remained significant after FDR correction. For lymphoma, although regular dairy consumption was significantly associated with 23% (1.23; 1.04–1.46) higher risk, the *P trend* value became non-significant after FDR correction. After correction for regression dilution bias, for each 50-g/day higher dairy consumption, the corresponding HRs were 1.07 (1.04–1.11) for total cancer, 1.12 (1.02–1.22) for liver cancer, 1.19 (1.01–1.41) for lymphoma and 1.17 (1.07–1.29) for breast cancer (Fig. 3). For other site-specific cancers, there were no clear associations with dairy consumption (Table 2).

**Table 2:** Adjusted hazard ratios for other site-specific cancers associated with dairy intake^a^

Types	Never/rarely intake		Monthly intake		Regular intake		*P* trend	FDR-corrected *P* trend^b^	*HR* (95% *CI*) per 50 g/day of usual dairy intake
	No. of cases	*HR* (95% *CI*)	No. of cases	*HR* (95% *CI*)	No. of cases	*HR* (95% *CI*)			
Oral cavity	573	1.00 (0.89–1.12)	98	0.97 (0.80–1.18)	156	0.98 (0.82–1.18)	0.83	0.90	1.00 (0.83–1.20)
Oesophagus	1953	1.00 (0.95–1.06)	209	0.89 (0.78–1.02)	306	1.07 (0.94–1.21)	0.73	0.90	1.03 (0.92–1.15)
Stomach	2495	1.00 (0.95–1.05)	338	1.06 (0.95–1.17)	744	1.08 (1.00–1.18)	0.10	0.34	1.07 (0.99–1.16)
Colon-rectum	2066	1.00 (0.94–1.06)	385	1.10 (1.00–1.21)	899	1.09 (1.01–1.18)	0.06	0.26	1.08 (1.00–1.17)
Pancreas	605	1.00 (0.89–1.12)	74	0.96 (0.76–1.20)	207	1.11 (0.95–1.31)	0.33	0.70	1.09 (0.93–1.29)
Larynx	142	1.00 (0.81–1.24)	19	0.79 (0.05–1.23)	48	1.08 (0.77–1.52)	0.86	0.90	1.05 (0.74–1.47)
Lung	4185	1.00 (0.96–1.04)	667	1.02 (0.94–1.10)	1430	1.03 (0.96–1.09)	0.48	0.82	1.03 (0.96–1.09)
Kidney	258	1.00 (0.85–1.18)	45	1.01 (0.76–1.35)	147	1.21 (0.99–1.48)	0.15	0.43	1.18 (0.96–1.46)
Bladder	368	1.00 (0.87–1.14)	66	1.04 (0.82–1.32)	134	0.84 (0.69–1.02)	0.19	0.46	0.91 (0.75–1.11)
Leukaemia	453	1.00 (0.88–1.13)	76	1.12 (0.89–1.39)	133	0.96 (0.79–1.18)	0.90	0.90	0.92 (0.75–1.13)
Prostate	278	1.00 (0.85–1.17)	40	0.93 (0.69–1.27)	107	0.95 (0.76–1.19)	0.70	0.90	0.97 (0.77–1.22)
Cervix	847	1.00 (0.91–1.09)	155	0.97 (0.83–1.14)	257	1.09 (0.93–1.26)	0.44	0.82	1.07 (0.92–1.25)
Endometrium	292	1.00 (0.85–1.17)	53	1.07 (0.82–1.40)	123	0.94 (0.76–1.16)	0.72	0.90	1.00 (0.80–1.25)
Ovary	324	1.00 (0.86–1.16)	54	0.91 (0.70–1.19)	129	0.98 (0.79–1.20)	0.79	0.90	1.03 (0.83–1.29)

*CI* confidence interval, *FDR* false discovery rate, *HR* hazard ratio

^a^Cox regression analyses were performed among 510,146 participants with no prior self-reported history of cancer at baseline. Analyses were stratified by age-at-risk (continuous variable), sex (dichotomous variable) and individual regions (ten regions) and were adjusted for education (four categories), income (four categories), smoking (four categories), alcohol consumption (four categories), total physical activity (continuous variable), family history of cancer (dichotomous variable), fresh fruit consumption (five categories), soy consumption (three categories) and body mass index (continuous variable)

^b^Significance was assessed at a 5% FDR

Associations of dairy consumption with risks of liver cancer, lymphoma and female breast cancer tended to be positive in nearly all of the subgroups, with few statistically significant heterogeneities being observed (*P* < 0.05, Additional file 1: Fig. 4-7), without adjusting for multiple testing. In particular, no significant heterogeneity was found across the five urban areas and five rural areas for all three types of cancers (Additional file 1: Fig. 7). Additionally, no significant heterogeneity was found between pre- and post-menopausal women for the association with breast cancer (Additional file 1: Fig. 6) and between men and women for the associations with any site-specific cancer (Additional file 1: Table 2).

The associations did not materially change in the various sensitivity analyses with further exclusions and further adjustments (Additional file 1: Table 4 and Additional file 1: Fig. 8-10).

## Discussion

In this large prospective study of Chinese adults with relatively low dairy consumption, higher dairy consumption was associated with a higher risk of overall cancer, liver cancer, female breast cancer and lymphoma, with each 50-g/day higher usual intake being associated with 7%, 12%, 17% and 19% higher risks, respectively; however, the association with lymphoma became non-significant after multiple-testing correction. The observed associations were independent of other lifestyle factors including adiposity and were largely consistent across the various subgroups of participants.

In recent decades, although dairy (mainly milk) consumption has increased substantially in China (mean intake from 14.9 g/day in 1992 to 24.7 g/day in 2012) [11], it is still much lower than in Western countries (e.g. mean intake of ~400 g/day in the USA in 2015) [25]. As in several previous surveys in China [14, 15], we observed higher consumption levels in urban than in rural areas, reflecting probably different stages of economic development in terms of availability and affordability [10, 14]. Our findings on the taller height at baseline and lower height loss during subsequent follow-up (i.e. from baseline to resurvey) in participants with higher dairy intake are also consistent with previous results from observational studies and randomized controlled trials [26, 27], supporting the role of dairy products in slowing down bone loss. The observed inverse association between dairy intake and body weight gain is also in line with some, but not all, previous reports [28], with calcium in dairy products potentially playing a role [29].

Liver cancer has been historically the most prevalent cancer type in China, with over half of the worldwide liver cancer incident cases and deaths being reported there [4]. This was chiefly due to the high prevalence of hepatitis B infection and aflatoxins exposure [4, 30] and the emerging roles of smoking and alcohol. Existing evidence linking dairy products to liver cancer is rather limited and inconsistent [31]. As in our study, the most recent meta-analysis of four prospective studies (1261 cases) from the USA and Europe reported a borderline significant ~30% higher risk of liver cancer comparing highest vs. lowest intake of dairy products [32]. Our study with 3191 cases added important new evidence linking dairy consumption with the risk of liver cancer, independent of hepatitis B infection.

Lymphoma is also one of the common cancers worldwide and in China, with chronic infections (e.g. Epstein-Barr virus) and certain occupational exposures to pesticides being the most well-established risk factors [33]. To date, four prospective studies, with 100–1300 cases each, have investigated the associations of dairy intake with risk of lymphoma [34–37], with none of them showing a significant association. However, in a meta-analysis including two of the aforementioned prospective studies [34, 36] and five case-control studies with a total of over 4000 cases, higher dairy intake was associated with 20% (2–42%) higher risk of lymphoma [38]. Our study provided further supportive evidence linking dairy consumption with the risk of lymphoma, although the causality of the association cannot be properly established.

Breast cancer is the most common type of cancer in women worldwide [1], accounting for 15% (~268,600 new cases) of all new cancers in China in 2015 [4]. According to the WCRF/AICR, the major risk factors for post-menopausal breast cancer include reproductive and hormonal factors, low physical activity, alcohol consumption and obesity [39]. The overall evidence on dairy intake in relation to both pre- and post-menopausal breast cancer, however, is limited [5, 39]. In one recent prospective study of 53,000 North-American women [23], dairy intake was positively associated with overall breast cancer risk, with a HR of 1.22 (1.05–1.40) comparing top vs. bottom deciles of dairy intake, broadly consistent with our study findings. This cohort included a high proportion of vegetarians and vegans with a much lower average dairy consumption compared to general Western populations [8, 40], similar to our population. This intake range is poorly investigated in previous studies, making it probably the only study to which our results could be well-compared.

Our study did not observe any significant association of dairy consumption with risks of other site-specific cancers, including colorectal and prostate cancer. Existing evidence tends to support an inverse association of dairy intake with colorectal cancer risk and a positive association with prostate cancer risk [5–7]. However, large heterogeneity by geographic region was observed in previous studies on the topic [5] and no significant association was reported from China on either dairy consumption [17] or dairy calcium intake in relation to risks of these two types of cancers [41]. Despite the recent increase, the incidence of colorectal and prostate cancers is still lower in China than in the West [4, 42, 43]. Studies involving a larger number of cases (e.g. prolonged follow-up of CKB) are needed to confirm (or refute) any modest associations.

Several biological mechanisms might possibly explain the positive findings in our study. Firstly, higher dairy intake may lead to higher plasma levels of insulin-like growth factor-I (IGF-I) [44, 45], which plays a key role in cellular proliferation and cancer development [44, 46, 47]. Higher levels of IGF-I have been associated with higher risks of several cancers including breast cancer, and recent Mendelian randomization studies have suggested that these associations could be causal [46–48]. Secondly, there is suggestive evidence that the relatively high content of branched-chain amino acids, lactose (which provides galactose), and IGF-I in milk could activate and enhance the mechanistic target of rapamycin complex 1 signalling, which might subsequently promote cell proliferation, leading potentially to carcinogenesis [49]. Thirdly, saturated fatty acids (SFA) and *trans*-fatty acids from dairy products have been associated with insulin resistance [50] and increased levels of proinflammatory cytokines [51], which are possible risk factors for the development of liver cancer [30, 31] and lymphoma [51], respectively. Fourthly, the observed association with breast cancer might relate to the fat-soluble sex hormones, such as oestrogen [52] and progesterone, contained in cow’s milk [53]. Finally, there is a hypothesis that people who are lactose intolerant may have altered ingestion of dairy products [54], which might produce different breakdown products and exert differences in risks of diseases, such as cancer [55]. However, there is still a large evidence gap and more studies are needed to explore further the potential mediating role of lactose intolerance in linking dairy intake and cancer risk.

The main strengths of our study include its prospective design, large sample size and long follow-up. We used repeat measurements in the resurvey to control regression dilution bias caused by long-term variation and measurement errors in the self-reported dairy intake. The rich data in CKB also allowed us to adjust for a wide range of potential confounders (including all other dietary variables) and to investigate the potential impacts of reverse causation. Nevertheless, our study has several limitations. Firstly, our questionnaire collected intake data only for few major food groups rather than for individual food items. Therefore, it was not possible to adjust for the intake of total energy or specific nutrients (e.g. SFA and calcium) or to distinguish the associations of individual dairy items (e.g. milk and cheese). Nonetheless, we have adjusted our analyses for both BMI and total physical activity, which together should provide a reasonable proxy for total energy intake [56–58], as the association between dairy consumption (mainly milk) and total energy intake in our study population is expected to be modest [59, 60]. Secondly, we could not assess the associations between different types of dairy products (i.e. milk, yoghurt and other dairy products) and cancer risk because such information was only collected during the second resurvey (with ~3 years of follow-up) of a subsample of participants (~24,700). Thirdly, we did not collect individual-level dairy consumption amount at baseline and the HRs for each 50 g/day of dairy consumption were estimated based on the assumption that the daily portions of dairy products did not change from baseline to the 2nd resurvey. Fourthly, although a large number of cancer cases were recorded, the statistical power remained low for certain less common cancer sites (e.g. prostate cancer). Even for common cancer types, the number of cases might not be large enough to allow reliable results in various subgroup analyses (e.g. by oestrogen receptor status in breast cancer). Lastly, although we adjusted analyses for a range of potential confounders, residual confounding may still exist; thus, the causality of the observed associations cannot be confirmed.

## Conclusions

In summary, in Chinese adults, higher dairy intake was associated with higher risks of liver cancer, lymphoma and female breast cancer. Our study was the first and largest Chinese prospective cohort study, which showed positive associations between dairy intake and risks of total and several site-specific cancers in China where the levels of dairy consumption are low but increasing. Future studies are warranted to establish the causality and potential underlying mechanisms involved. The findings of this study in combination with future studies might provide important information for evidence-based dietary recommendations on cancer prevention in China.

## Supplementary Information

**Additional file 1: **Methods. Calculation of the usual amount of dairy intake and regression dilution bias correction. **Table 1**. Baseline and usual amount (g/day) for each baseline category of dairy consumption based on 24,700* participants who attended the second resurvey in 2013-2014. **Table 2**. Incidence rates of cancers (ICD-10 coded) at 30-79 years and adjusted HRs (95% CIs) for cancers per 50 g/day of usual dairy intake in men and women from CKB, without prior self-reported history of cancer at baseline. **Table 3**. Female reproductive characteristics by frequency of dairy intake at baseline (2004-2008). **Table 4**. Adjusted HRs for cancer risk associated with dairy intake in sensitivity analyses. **Figure 1**. Percentage of regular dairy consumers by sex and survey region at baseline (2004-2008). **Figure 2**. Estimated mean dairy intake (g/day) by sex and survey region at baseline (2004-2008). **Figure 3**. Sex-specific changes in standing height, body weight and body mass index between baseline (2004-2008) and second resurvey (2013-2014) by frequency of baseline dairy intake (n = 18,132). **Figure 4**. Adjusted HRs (95% CIs) for liver cancer per 50 g/day of usual dairy intake by baseline characteristics. **Figure 5**. Adjusted HRs (95% CIs) for lymphoma per 50 g/day of usual dairy intake by baseline characteristics. **Figure 6**. Adjusted HRs (95% CIs) for female breast cancer per 50 g/day of usual dairy intake by baseline characteristics. **Figure 7**. Adjusted HRs (95% CIs) for liver cancer, lymphoma and female breast cancer per 50 g/day of usual dairy intake by region. **Figure 8**. Adjusted HRs (95% CIs) for liver cancer per 50 g/day of usual dairy intake, with step-wise adjustment. **Figure 9**. Adjusted HRs (95% CIs) for lymphoma per 50 g/day of usual dairy intake, with step-wise adjustment. **Figure 10**. Adjusted HRs (95% CIs) for female breast cancer per 50 g/day of usual dairy intake, with step-wise adjustment.

## Data Availability

The China Kadoorie Biobank (CKB) is a global resource for the investigation of lifestyle, environmental, blood biochemical and genetic factors as determinants of common diseases. The CKB study group is committed to making the cohort data available to the scientific community in China, the UK and worldwide to advance knowledge about the causes, prevention and treatment of disease. For detailed information on what data is currently available to open access users and how to apply for it, visit http://www.ckbiobank.org/site/Data+Access. Researchers who are interested in obtaining the raw data from the China Kadoorie Biobank study that underlines this paper should contact ckbaccess@ndph.ox.ac.uk. A research proposal will be requested to ensure that any analysis is performed by bona fide researchers and — where data is not currently available to open access researchers — is restricted to the topic covered in this paper.

## Abbreviations

BF%: Body fat percentage
BMI: Body mass index
CI: Confidence interval
CKB: China Kadoorie Biobank
FDR: False discovery rate
HBsAg: Hepatitis B surface antigen
HR: Hazard ratio
ICD: International Classification of Diseases
IGF-I: Insulin-like growth factor-I
SD: Standard deviation
SFA: Saturated fatty acids
WC: Waist circumference
WCRF/AICR: World Cancer Research Fund/American Institute for Cancer Research
