# Supplementary material for: Dairy consumption and risks of total and site-specific cancers in Chinese adults: an 11-year prospective study of 0.5 million people
Source: BMC Med. 2022 May 6;20:134. doi: 10.1186/s12916-022-02330-3 (PMC9074208; doi:10.1186/s12916-022-02330-3)
Supplement: Supplementary file 1 — Additional file 1: Methods. Calculation of the usual amount of dairy intake and regression dilution bias correction. Table 1. Baseline and usual amount (g/day) for each baseline category of dairy consumption based on 24,700* participants who attended the second resurvey in 2013-2014. Table 2. Incidence rates of cancers (ICD-10 coded) at 30-79 years and adjusted HRs (95% CIs) for cancers per 50 g/day of usual dairy intake in men and women from CKB, without prior self-reported history of cancer at baseline. Table 3. Female reproductive characteristics by frequency of dairy intake at baseline (2004-2008). Table 4. Adjusted HRs for cancer risk associated with dairy intake in sensitivity analyses. Figure 1. Percentage of regular dairy consumers by sex and survey region at baseline (2004-2008). Figure 2. Estimated mean dairy intake (g/day) by sex and survey region at baseline (2004-2008). Figure 3. Sex-specific changes in standing height, body weight and body mass index between baseline (2004-2008) and second resurvey (2013-2014) by frequency of baseline dairy intake (n = 18,132). Figure 4. Adjusted HRs (95% CIs) for liver cancer per 50 g/day of usual dairy intake by baseline characteristics. Figure 5. Adjusted HRs (95% CIs) for lymphoma per 50 g/day of usual dairy intake by baseline characteristics. Figure 6. Adjusted HRs (95% CIs) for female breast cancer per 50 g/day of usual dairy intake by baseline characteristics. Figure 7. Adjusted HRs (95% CIs) for liver cancer, lymphoma and female breast cancer per 50 g/day of usual dairy intake by region. Figure 8. Adjusted HRs (95% CIs) for liver cancer per 50 g/day of usual dairy intake, with step-wise adjustment. Figure 9. Adjusted HRs (95% CIs) for lymphoma per 50 g/day of usual dairy intake, with step-wise adjustment. Figure 10. Adjusted HRs (95% CIs) for female breast cancer per 50 g/day of usual dairy intake, with step-wise adjustment. [file 12916_2022_2330_MOESM1_ESM.docx]

**Dairy consumption and risks of total and site-specific cancers in Chinese adults: an 11-year prospective study of 0.5 million people**

**Additional File 1**

**Table of Contents**

[Additional file 1: Methods. Calculation of the usual amount of dairy intake and regression dilution bias correction 2](#_Toc94700074)

[Additional file 1: Table 1. Baseline and usual amount (g/day) for each baseline category of dairy consumption based on 24,700* participants who attended the second resurvey in 2013-2014 3](#_Toc94700075)

[Additional file 1: Table 2. Incidence rates of cancers (ICD-10 coded) at 30-79 years and adjusted HRs (95% CIs) for cancers per 50 g/day of usual dairy intake in men and women from CKB, without prior self-reported history of cancer at baseline 4](#_Toc94700076)

[Additional file 1: Table 3. Female reproductive characteristics by frequency of dairy intake at baseline (2004-2008) 5](#_Toc94700077)

[Additional file 1: Table 4. Adjusted HRs for cancer risk associated with dairy intake in sensitivity analyses 6](#_Toc94700078)

[Additional file 1: Fig. 1. Percentage of regular dairy consumers by sex and survey region at baseline (2004-2008) 7](#_Toc94700079)

[Additional file 1: Fig. 2. Estimated mean dairy intake (g/day) by sex and survey region at baseline (2004-2008) 8](#_Toc94700080)

[Additional file 1: Fig. 3. Sex-specific changes in standing height, body weight and body mass index between baseline (2004-2008) and second resurvey (2013-2014) by frequency of baseline dairy intake (n = 18,132). 9](#_Toc94700081)

[Additional file 1: Fig. 4. Adjusted HRs (95% CIs) for liver cancer per 50 g/day of usual dairy intake by baseline characteristics. 10](#_Toc94700082)

[Additional file 1: Fig. 5. Adjusted HRs (95% CIs) for lymphoma per 50 g/day of usual dairy intake by baseline characteristics. 11](#_Toc94700083)

[Additional file 1: Fig. 6. Adjusted HRs (95% CIs) for female breast cancer per 50 g/day of usual dairy intake by baseline characteristics. 12](#_Toc94700084)

[Additional file 1: Fig. 7. Adjusted HRs (95% CIs) for liver cancer, lymphoma and female breast cancer per 50 g/day of usual dairy intake by region. 13](#_Toc94700085)

[Additional file 1: Fig. 8. Adjusted HRs (95% CIs) for liver cancer per 50 g/day of usual dairy intake, with step-wise adjustment. 14](#_Toc94700086)

[Additional file 1: Fig. 9. Adjusted HRs (95% CIs) for lymphoma per 50 g/day of usual dairy intake, with step-wise adjustment. 15](#_Toc94700087)

[Additional file 1: Fig. 10. Adjusted HRs (95% CIs) for female breast cancer per 50 g/day of usual dairy intake, with step-wise adjustment. 16](#_Toc94700088)

# Additional file 1: Methods. Calculation of the usual amount of dairy intake and regression dilution bias correction

The mean usual amount of dairy consumption (g/day) for each baseline consumption frequency category was calculated using consumption frequency data collected at the baseline survey combined with frequency and amount data collected at the second resurvey. The second resurvey was conducted in 2013-2014 among a randomly selected subsample of surviving CKB participants without cancer and missing BMI values at either baseline or second resurvey (n=24,700).

During the second resurvey, the consumption frequency and daily portion of dairy products was collected separately for three types of dairy products, i.e. milk, yoghurt and other dairy products (e.g. cheese and milk powder). For calculating the usual amount of total dairy products (g/day) consumed by each participant, the following steps were followed:

1. Milk consumption amount (ml) was converted into grams using the formula “milk in ml*1.03”, where 1.03 is the average density of liquid milk (whole, skimmed, partially skimmed) at 20^0^C.
2. Using the data collected in the second resurvey, for each type of dairy products, the mean daily portion in grams was derived using the reported mean daily consumption amount for each of the five dairy consumption categories, multiplied by the mean number of days per month consuming dairy products* and divided by 30. *The mean number of days per month assigned to each group was 0 for the ‘never/rarely’ group, 2.5 days for the ‘monthly’ group, 8.6 days for the ‘1-3 days/week’ group, 21.5 days for the ‘4-6 days/week’ group and 30 days for the ‘daily’ group.
3. The intake amount (g/day) of total dairy products was calculated as the sum of the daily portion in grams from all three dairy products.
4. The mean consumption amount of total dairy products (g/day) for each category, i.e. 143.4 g/day for daily consumption group, 101.0 for 4-6 days/week group, 84.4 for1-3 days/week group, 48.1 for monthly group and 0 g/day for never/rarely group, respectively, was assigned to each individual participant in the corresponding baseline group as the proxy for baseline amount of consumption (**B,** **eTable1**).
5. The average usual amount of dairy consumption (**U**) for each baseline frequency category was then calculated by multiplying **B** by the percentage of participants in each of the baseline consumption frequency group at the second resurvey. For instance, for the baseline ‘never/rarely’ group, at the resurvey 73.7% remained at the ‘never/rarely’ group, 7.8% switched to the ‘monthly’ group, 9.2% switched to the ‘1-3 days per week’ group, 2.0% switched to the ‘4-6 days per week’ group, and 7.3% switched to the ‘daily’ group. The usual amount of dairy consumption for the baseline ‘never/rarely’ group was therefore equal to 73.7%*0 + 7.8%*48.1 + 9.2%*84.4 + 2.0%*101.0 + 7.3%*143.4 = 24.0 g/day. By using the same approach, the usual amount of dairy consumption for the other baseline categories of consumption (i.e. monthly, 1-3 days/week, 4-6 days/week and daily) were calculated as 44.4, 68.4, 77.2, 92.4 g/day, respectively (eTable1).

# Additional file 1: Table 1. Baseline and usual amount (g/day) for each baseline category of dairy consumption based on 24,700* participants who attended the second resurvey in 2013-2014

| **Percentage (%)†**  **Baseline** | | **Second resurvey** | | | | | **Baseline consumption (g/day)** | **Usual consumption (g/day) ‡** |
| --- | --- | --- | --- | --- | --- | --- | --- | --- |
|  |  | Never/rarely  1 | Monthly  2 | 1-3 d/wk  3 | 4-6 d/wk  4 | Daily  5 |  |  |
|  |  |  |  |  |  |  | **B** | **U** |
| 1 | Never/rarely | 73.7% | 7.8% | 9.2% | 2.0% | 7.3% | B1 = 0 | U1 = 24.0 |
|  |  | (12952) | (1376) | (1624) | (349) | (1285) |  |  |
| 2 | Monthly | 53.4% | 11.1% | 17.8% | 3.4% | 14.4% | B2 = 48.1 | U2 = 44.4 |
|  |  | (1402) | (290) | (467) | (88) | (378) |  |  |
| 3 | 1-3 d/wk | 33.3% | 9.5% | 25.8% | 6.8% | 24.5% | B3 = 84.4 | U3 = 68.4 |
|  |  | (636) | (182) | (492) | (130) | (468) |  |  |
| 4 | 4-6 d/wk | 29.6% | 5.8% | 25.7% | 7.3% | 31.7% | B4 = 101.1 | U4 = 77.2 |
|  |  | (143) | (28) | (124) | (35) | (153) |  |  |
| 5 | Daily | 23.7% | 5.0% | 17.2% | 5.2% | 49.1% | B5 = 143.4 | U5 = 92.4 |
|  |  | (497) | (104) | (360) | (108) | (1029) |  |  |

* The 24,700 participants were a randomly selected subsample of surviving CKB participants without cancer and missing BMI values at either baseline or second resurvey.

† The percentage of each frequency level in the second resurvey was calculated according to five frequency levels in the baseline survey.

‡ Usual intake amount for each group was estimated by taking into account changes in consumption frequency between baseline and second resurvey using this formula U_n_ = $\sum_{i=1}^{5} (F\mathrm{ni} \times Bi)$; F is the percentage in each cell, B is the baseline consumption amount (g/day) for each baseline category, U is the usual consumption amount (g/day) for each baseline category.

# Additional file 1: Table 2. Incidence rates of cancers (ICD-10 coded) at 30-79 years and adjusted HRs (95% CIs) for cancers per 50 g/day of usual dairy intake in men and women from CKB, without prior self-reported history of cancer at baseline

| **Cancer types** | **ICD-10 codes** | **Incidence rate per 1000 person-years** | **Cancer types** | **Incidence rate per 1000 person-years** | **No. of cases**† | **HR (95% CI) per 50 g/day of usual dairy intake**‡ | **Cancer types** | **Incidence rate per 1000 person-years** | **No. of cases**† | **HR (95% CI) per 50 g/day of usual dairy intake**‡ |
| --- | --- | --- | --- | --- | --- | --- | --- | --- | --- | --- |
|  | **Total (n = 510,146)** | | **Men (n = 209,235)** | | | | **Women (n = 300,911)** | | | |
| All cancers | C00-C97 | 5.47 | All cancers | 6.63 | 14261 | 1.03 (0.99-1.08) | All cancers | 4.69 | 15016 | 1.10 (1.06-1.15) |
| Lung | C33-C34 | 1.16 | Lung | 1.72 | 3735 | 0.98 (0.90-1.06) | Breast | 0.80 | 2582 | 1.17 (1.07-1.29) |
| Female Breast | C50 | 0.80 | Stomach | 1.09 | 2366 | 1.06 (0.96-1.17) | Lung | 0.79 | 2547 | 1.09 (0.99-1.20) |
| Stomach | C16 | 0.66 | Liver | 0.96 | 2055 | 1.09 (0.97-1.21) | Colon-rectum | 0.52 | 1702 | 1.07 (0.95-1.20) |
| Colon-rectum | C18-C20 | 0.62 | Oesophagus | 0.78 | 1698 | 0.98 (0.86-1.13) | Cervix | 0.39 | 1259 | 1.07 (0.92-1.25) |
| Liver | C22 | 0.60 | Colon-rectum | 0.76 | 1648 | 1.10 (0.98-1.23) | Stomach | 0.37 | 1211 | 1.09 (0.95-1.26) |
| Oesophagus | C15 | 0.46 | Oral cavity | 0.23 | 501 | 0.94 (0.73-1.20) | Liver | 0.36 | 1136 | 1.18 (1.02-1.37) |
| Cervix | C53 | 0.39 | Lymphoma | 0.21 | 447 | 1.04 (0.80-1.35) | Oesophagus | 0.24 | 770 | 1.15 (0.94-1.40) |
| Prostate | C61 | 0.20 | Pancreas | 0.20 | 441 | 1.07 (0.85-1.35) | Ovary | 0.16 | 507 | 1.03 (0.83-1.29) |
| Lymphoma | C81-C85 | 0.17 | Prostate | 0.20 | 425 | 0.97 (0.77-1.22) | Pancreas | 0.14 | 445 | 1.13 (0.89-1.42) |
| Pancreas | C25 | 0.16 | Bladder | 0.18 | 398 | 0.95 (0.75-1.20) | Lymphoma | 0.14 | 468 | 1.33 (1.06-1.67) |
| Ovary | C56 | 0.16 | Leukaemia | 0.14 | 313 | 0.94 (0.70-1.26) | Endometrium | 0.14 | 468 | 1.00 (0.80-1.25) |
| Oral cavity | C00-C14 | 0.15 | Kidney | 0.11 | 231 | 1.35 (1.01-1.81) | Leukaemia | 0.11 | 349 | 0.90 (0.69-1.19) |
| Endometrium | C54.1 | 0.14 | Larynx | 0.08 | 172 | 1.15 (0.79-1.68) | Oral cavity | 0.10 | 326 | 1.07 (0.81-1.41) |
| Leukaemia | C91-C95 | 0.12 |  |  |  |  | Kidney | 0.07 | 219 | 1.04 (0.77-1.41) |
| Bladder | C67 | 0.10 |  |  |  |  | Bladder | 0.05 | 170 | 0.83 (0.57-1.19) |
| Kidney | C64 | 0.08 |  |  |  |  | Larynx | 0.01 | 37 | 0.71 (0.32-1.59) |
| Larynx | C32 | 0.04 |  |  |  |  |  |  |  |  |

CI = confidence interval. HR = hazard ratio. ICD-10 = International Classification of Diseases-10

†No of cases refers to the number of incident cancer cases in each group.

‡Cox regression analyses were stratified by age-at-risk (continuous variable), sex (dichotomous variable) and individual regions (ten regions) and were adjusted for education (four categories), income (four categories), smoking (four categories), alcohol consumption (four categories), total physical activity (continuous variable), family history of cancer (dichotomous variable), fresh fruit consumption (five categories), soy consumption (three categories) and body mass index (continuous variable). Analysis of liver cancer was additionally adjusted for Hepatitis B surface antigen status (three categories). The p-het values for the heterogeneity of the associations of dairy intake with cancer risk among the two sexes were all >0.05.

# Additional file 1: Table 3. Female reproductive characteristics by frequency of dairy intake at baseline (2004-2008)

| Female reproductive health characteristics | Frequency of dairy intake | | | Overall  n = 300,911^a^ |
| --- | --- | --- | --- | --- |
|  | **Never/rarely** | **Monthly** | **Regular** |  |
|  | **n = 204,180** | **n = 32,782** | **n = 63,949** |  |
| Postmenopausal women, % | 56.8 | 58.9 | 57.9 | 57.3 |
| Ever use of oral contraceptives, % | 9.6 | 9.9 | 10.6 | 9.8 |
| Mean age at menarche (SD), years | 15.5 (2.0) | 15.4 (1.9) | 15.3 (2.1) | 15.4 (2.0) |
| Mean number of children (SD) | 2.2 (1.0) | 2.1 (0.9) | 2.1 (1.1) | 2.1 (1.3) |
| Mean age at first birth (SD), years | 23.4 (2.9) | 23.4 (2.7) | 23.4 (3.1) | 23.4 (3.2) |
| Mean breastfeeding duration (SD), months^b^ | 14.8 (6.8) | 14.3 (6.5) | 14.1 (7.5) | 14.6 (7.6) |
| Mean age at menopause (SD), years^c^ | 48.2 (4.4) | 48.2 (4.2) | 48.3 (4.7) | 48.2 (4.4) |

SD = standard deviation.

Multiple linear regression (for continuous outcomes) or logistic regression (for binary outcomes) were used to calculate the means (SDs) or percentages of various baseline characteristics across three frequency categories of dairy consumption (i.e. never/rarely, monthly and ≥1 day/week-characterised as regular), with adjustments for age (continuous variable), region (ten regions), education (four categories) and income (four categories).

^a^There were in total 300,911 female participants included in this study; 47 of them had missing values for menopausal status, oral contraceptives use and age at menarche; 4105 of them had missing values for age at first birth and breastfeeding duration.

^b^Mean was calculated from the total breastfeeding duration, which was derived from the breastfeeding of all children.

^c^Mean was calculated from 157,586 post-menopausal women, who self-reported age at menopause.

# Additional file 1: Table 4. Adjusted HRs for cancer risk associated with dairy intake in sensitivity analyses

| **Cancer type** | **Never/rarely intake** | | **Monthly intake** | | **Regular intake** | |
| --- | --- | --- | --- | --- | --- | --- |
|  | No. of cases | HR (95% CI) | No. of cases | HR (95% CI) | No. of cases | HR (95% CI) |
| **Additional adjustment for prevalent cardiovascular disease at baseline** | | | | | | |
| All cancers | 19340 | 1.00 (0.98, 1.02) | 3120 | 1.03 (1.00, 1.07) | 6817 | 1.09 (1.06, 1.12) |
| Liver^a^ | 2171 | 1.00 (0.95, 1.06) | 335 | 1.01 (0.90, 1.12) | 685 | 1.18 (1.08, 1.29) |
| Lymphoma | 615 | 1.00 (0.90, 1.11) | 108 | 1.21 (1.01, 1.46) | 192 | 1.22 (1.03, 1.45) |
| Breast | 1354 | 1.00 (0.93, 1.07) | 321 | 1.13 (1.02, 1.26) | 907 | 1.21 (1.12, 1.32) |
| **Additional adjustment for prevalent diabetes at baseline** | | | | | | |
| All cancers | 19340 | 1.00 (0.98, 1.02) | 3120 | 1.03 (1.00, 1.07) | 6817 | 1.08 (1.05, 1.11) |
| Liver^a^ | 2171 | 1.00 (0.95, 1.06) | 335 | 1.00 (0.90, 1.11) | 685 | 1.15 (1.05, 1.25) |
| Lymphoma | 615 | 1.00 (0.90, 1.11) | 108 | 1.22 (1.01, 1.47) | 192 | 1.23 (1.04, 1.46) |
| Breast | 1354 | 1.00 (0.93, 1.07) | 321 | 1.13 (1.01, 1.26) | 907 | 1.21 (1.11, 1.31) |
| **Additional adjustment for standing height** | | | | | | |
| All cancers | 19340 | 1.00 (0.98, 1.02) | 3120 | 1.03 (0.99, 1.07) | 6817 | 1.08 (1.05, 1.11) |
| Liver^a^ | 2171 | 1.00 (0.95, 1.06) | 335 | 1.00 (0.90, 1.12) | 685 | 1.18 (1.08, 1.28) |
| Lymphoma | 615 | 1.00 (0.90, 1.11) | 108 | 1.21 (1.00, 1.45) | 192 | 1.21 (1.02, 1.44) |
| Breast | 1354 | 1.00 (0.93, 1.07) | 321 | 1.12 (1.01, 1.25) | 907 | 1.20 (1.10, 1.30) |
| **Additional adjustment for waist circumference and body fat percentage** | | | | | | |
| All cancers | 19330 | 1.00 (0.98, 1.02) | 3119 | 1.03 (0.99, 1.07) | 6815 | 1.08 (1.05, 1.12) |
| Liver^a^ | 2167 | 1.00 (0.95, 1.06) | 335 | 1.00 (0.90, 1.11) | 685 | 1.17 (1.07, 1.28) |
| Lymphoma | 615 | 1.00 (0.90, 1.11) | 108 | 1.21 (1.00, 1.45) | 192 | 1.22 (1.03, 1.45) |
| Breast | 1354 | 1.00 (0.93, 1.07) | 321 | 1.13 (1.01, 1.26) | 907 | 1.22 (1.12, 1.32) |
| **Additional adjustment for red meat, poultry, fish and eggs** | | | | | | |
| All cancers | 19340 | 1.00 (0.98, 1.02) | 3120 | 1.03 (1.00, 1.07) | 6817 | 1.08 (1.05, 1.12) |
| Liver^a^ | 2171 | 1.00 (0.95, 1.06) | 335 | 1.01 (0.91, 1.12) | 685 | 1.18 (1.08, 1.29) |
| Lymphoma | 615 | 1.00 (0.90, 1.11) | 108 | 1.21 (1.00, 1.46) | 192 | 1.24 (1.05, 1.47) |
| Breast | 1354 | 1.00 (0.93, 1.07) | 321 | 1.13 (1.02, 1.26) | 907 | 1.22 (1.12, 1.32) |
| **Additional adjustment for fresh and preserved vegetables and coarse grain foods** | | | | | | |
| All cancers | 19340 | 1.00 (0.98, 1.02) | 3120 | 1.03 (1.00, 1.07) | 6817 | 1.08 (1.05, 1.12) |
| Liver^a^ | 2171 | 1.00 (0.95, 1.06) | 335 | 1.00 (0.89, 1.11) | 685 | 1.17 (1.07, 1.28) |
| Lymphoma | 615 | 1.00 (0.90, 1.11) | 108 | 1.24 (1.03, 1.49) | 192 | 1.24 (1.05, 1.48) |
| Breast | 1354 | 1.00 (0.93, 1.07) | 321 | 1.12 (1.01, 1.25) | 907 | 1.21 (1.12, 1.32) |
| **Additional adjustment for rice and wheat foods** | | | | | | |
| All cancers | 19340 | 1.00 (0.98, 1.02) | 3120 | 1.03 (1.00, 1.07) | 6817 | 1.09 (1.05, 1.12) |
| Liver^a^ | 2171 | 1.00 (0.95, 1.06) | 335 | 1.01 (0.91, 1.12) | 685 | 1.18 (1.08, 1.29) |
| Lymphoma | 615 | 1.00 (0.90, 1.11) | 108 | 1.21 (1.00, 1.46) | 192 | 1.22 (1.03, 1.45) |
| Breast | 1354 | 1.00 (0.93, 1.07) | 321 | 1.13 (1.02, 1.26) | 907 | 1.21 (1.12, 1.31) |
| **Exclusion of the first 2 years of follow-up** | | | | | | |
| All cancers | 16569 | 1.00 (0.98, 1.02) | 2653 | 1.01 (0.97, 1.05) | 5844 | 1.08 (1.04, 1.11) |
| Liver^a^ | 1809 | 1.00 (0.94, 1.06) | 272 | 0.94 (0.84, 1.06) | 578 | 1.16 (1.05, 1.28) |
| Lymphoma | 545 | 1.00 (0.89, 1.12) | 96 | 1.20 (0.98, 1.46) | 164 | 1.19 (0.99, 1.43) |
| Breast | 1193 | 1.00 (0.93, 1.08) | 276 | 1.11 (0.99, 1.25) | 802 | 1.25 (1.14, 1.36) |
| **Exclusion of participants with baseline age <35 or >74 years^b^** | | | | | | |
| All cancers | 18976 | 1.00 (0.98, 1.02) | 3068 | 1.04 (1.00, 1.08) | 6651 | 1.09 (1.06, 1.13) |
| Liver^a^ | 2127 | 1.00 (0.95, 1.06) | 330 | 1.02 (0.91, 1.13) | 668 | 1.20 (1.09, 1.31) |
| Lymphoma | 611 | 1.00 (0.90, 1.11) | 107 | 1.21 (1.00, 1.46) | 189 | 1.22 (1.03, 1.45) |
| Breast | 1334 | 1.00 (0.93, 1.07) | 314 | 1.13 (1.01, 1.26) | 892 | 1.22 (1.13, 1.33) |
| **Additional adjustment for prevalent chronic hepatitis/cirrhosis at baseline** | | | | | | |
| Liver^a^ | 2171 | 1.00 (0.95, 1.06) | 335 | 1.00 (0.90, 1.11) | 685 | 1.16 (1.06, 1.27) |
| **Additional adjustment for occupation** | | | | | | |
| Lymphoma | 615 | 1.00 (0.90, 1.11) | 108 | 1.21 (1.01, 1.46) | 192 | 1.22 (1.03, 1.45) |
| **Additional adjustment for female reproductive factors^c^** | | | | | | |
| Breast | 1337 | 1.00 (0.93, 1.07) | 314 | 1.12 (1.00, 1.25) | 879 | 1.19 (1.09, 1.29) |
| **Additional adjustment for age at menopause^d^** | | | | | | |
| Breast | 649 | 1.00 (0.91, 1.10) | 158 | 1.16 (0.99, 1.35) | 484 | 1.26 (1.13, 1.41) |
| **Exclusion of women with history of lumpectomy at baseline** | | | | | | |
| Breast | 1306 | 1.00 (0.93, 1.07) | 310 | 1.13 (1.01, 1.26) | 868 | 1.21 (1.11, 1.31) |

CI = confidence interval. HR = hazard ratio.

Cox regression analyses was performed among 510,146 participants with no prior self-reported history of cancer at baseline. Main analyses were stratified by age-at-risk (continuous variable), sex (dichotomous variable) and region (ten regions) and were adjusted for education (four categories), income (four categories), smoking (four categories), alcohol consumption (four categories), total physical activity (continuous variable), family history of cancer (dichotomous variable), fresh fruit consumption (five categories), soy consumption (three categories) and BMI (continuous variable).

^a^Main analysis of liver cancer was additionally adjusted for hepatitis B surface antigen status (three categories).

^b^Among the total of 510,146 participants included in the current analysis, a total of 17,010 participants were excluded (9812 and 7198 with baseline age below 35 and above 74 years, respectively).

^c^Female reproductive factors include age at menarche (continuous variable), age at first birth (continuous variable), number of children (continuous variable), breastfeeding duration (continuous variable), use of oral contraceptives (dichotomous variable), and menopausal status (dichotomous variable).

^d^Including post-menopausal women only.

Additional file 1: Fig. 1. Percentage of regular dairy consumers by sex and survey region at baseline (2004-2008)

Values were adjusted for age (continuous variable). Bars with grey diagonal lines indicate urban study areas and solid dark grey bars indicate rural study areas.

Additional file 1: Fig. 2. Estimated mean dairy intake (g/day) by sex and survey region at baseline (2004-2008)

Values were adjusted for age (continuous variable). Bars with grey diagonal lines indicate urban study areas and solid dark grey bars indicate rural study areas.

Additional file 1: Fig. 3. Sex-specific changes in standing height, body weight and body mass index between baseline (2004-2008) and second resurvey (2013-2014) by frequency of baseline dairy intake (n = 18,132).

Analyses included participants (n = 18,132), who did not report history of cancer, cardiovascular disease and diabetes at either baseline or second resurvey and with no incident cancer, cardiovascular disease and diabetes before 31 July 2013 (before the start of second resurvey). Linear regression analyses were adjusted for the study year interval between baseline and second resurvey (continuous variable), region (ten regions), and for baseline age (continuous variable), education (four categories), income (four categories), smoking (four categories), alcohol consumption (four categories), total physical activity (continuous variable), fresh fruit consumption (five categories), standing height (continuous variable) and body weight (continuous variable). Vertical lines represent 95% CIs. Solid squares represent men and open squares represent women.

Additional file 1: Fig. 4. Adjusted HRs (95% CIs) for liver cancer per 50 g/day of usual dairy intake by baseline characteristics.

Cox regression analyses were stratified by age-at-risk (continuous variable), sex (dichotomous variable) and region (ten regions) and were adjusted for education (four categories), income (four categories), smoking (four categories), alcohol consumption (four categories), total physical activity (continuous variable), family history of cancer (dichotomous variable), fresh fruit consumption (five categories), soy consumption (three categories), BMI (continuous variable) and hepatitis B surface antigen status (three categories). ^a^Overall HR per 50 g/day usual dairy intake after correcting for regression dilution bias. ^b^Overall HR per 50 g/day baseline dairy intake before correcting for regression dilution bias. Black squares, HRs (size is inversely proportional to the variance of the log_e_ of HR); horizontal lines represent 95% CIs; white diamonds, overall HRs (95% CIs); ‘No of cases’ refers to the number of incident cancer cases in each group; the subscript numbers in the chi-square values represent the degrees of freedom.

Additional file 1: Fig. 5. Adjusted HRs (95% CIs) for lymphoma per 50 g/day of usual dairy intake by baseline characteristics.

Cox regression analyses were stratified by age-at-risk (continuous variable), sex (dichotomous variable) and region (ten regions) and were adjusted for education (four categories), income (four categories), smoking (four categories), alcohol consumption (four categories), total physical activity (continuous variable), family history of cancer (dichotomous variable), fresh fruit consumption (five categories), soy consumption (three categories) and BMI (continuous variable). ^a^Overall HR per 50 g/day usual dairy intake after correcting for regression dilution bias. ^b^Overall HR per 50 g/day baseline dairy intake before correcting for regression dilution bias. Black squares, HRs (size is inversely proportional to the variance of the log_e_ of HR); horizontal lines represent 95% CIs; white diamonds, overall HRs (95% CIs); ‘No of cases’ refers to the number of incident cancer cases in each group; the subscript numbers in the chi-square values represent the degrees of freedom.

Additional file 1: Fig. 6. Adjusted HRs (95% CIs) for female breast cancer per 50 g/day of usual dairy intake by baseline characteristics.

Cox regression analyses were stratified by age-at-risk (continuous variable), sex (dichotomous variable) and region (ten regions) and were adjusted for education (four categories), income (four categories), smoking (four categories), alcohol consumption (four categories), total physical activity (continuous variable), family history of cancer (dichotomous variable), fresh fruit consumption (five categories), soy consumption (three categories) and BMI (continuous variable). Menopausal status was self-reported at baseline. ^a^Overall HR per 50 g/day usual dairy intake after correcting for regression dilution bias. ^b^Overall HR per 50 g/day baseline dairy intake before correcting for regression dilution bias. Black squares, HRs (size is inversely proportional to the variance of the log_e_ of HR); horizontal lines represent 95% CIs; white diamonds, overall HRs (95% CIs); ‘No of cases’ refers to the number of incident cancer cases in each group; the subscript numbers in the chi-square values represent the degrees of freedom.

Additional file 1: Fig. 7. Adjusted HRs (95% CIs) for liver cancer, lymphoma and female breast cancer per 50 g/day of usual dairy intake by region.

Cox regression analyses were stratified by age-at-risk (continuous variable), sex (dichotomous variable) and region (ten regions) and were adjusted for education (four categories), income (four categories), smoking (four categories), alcohol consumption (four categories), total physical activity (continuous variable), family history of cancer (dichotomous variable), fresh fruit consumption (five categories), soy consumption (three categories) and BMI (continuous variable). Analysis for liver cancer was additionally adjusted for hepatitis B surface antigen status (three categories). Overall HR per 50 g/day usual dairy intake after correcting for regression dilution bias. Black squares, HRs (size is inversely proportional to the variance of the log_e_ of HR); horizontal lines represent 95% CIs; white diamonds, overall HRs (95% CIs); ‘No of cases’ refers to the number of incident cancer cases in each group; the subscript numbers in the chi-square values represent the degrees of freedom.

Additional file 1: Fig. 8. Adjusted HRs (95% CIs) for liver cancer per 50 g/day of usual dairy intake, with step-wise adjustment.

The likelihood ratio chi-squared values indicate the strength of the associations of dairy intake with liver cancer. A larger chi-squared value indicates a stronger association and a smaller chi-squared value indicates that the association is attenuated after additionally adjusting for the newly added variable. *Basic Cox model was stratified by age-at-risk (continuous variable), sex (dichotomous variable) and region (ten regions) and was adjusted for education (four categories).

Additional file 1: Fig. 9. Adjusted HRs (95% CIs) for lymphoma per 50 g/day of usual dairy intake, with step-wise adjustment.

The likelihood ratio chi-squared values indicate the strength of the associations of dairy intake with lymphoma. A larger chi-squared value indicates a stronger association and smaller chi-squared value indicates that the association is attenuated after additionally adjusting for the newly added variable. *Basic Cox model was stratified by age-at-risk (continuous variable), sex (dichotomous variable) and region (ten regions) and was adjusted for education (four categories).

Additional file 1: Fig. 10. Adjusted HRs (95% CIs) for female breast cancer per 50 g/day of usual dairy intake, with step-wise adjustment.

The likelihood ratio chi-squared values indicate the strength of the associations of dairy intake with female breast cancer. A larger chi-squared value indicates a stronger association and smaller chi-squared value indicates that the association is attenuated after additionally adjusting for the newly added variable. *Basic Cox model was stratified by age-at-risk (continuous variable), sex (dichotomous variable) and region (ten regions) and was adjusted for education (four categories).
